# Genetic and Phenotypic Characterization of *Botrytis* Populations from Economic and Wild Host Plants in Iran

**DOI:** 10.3390/jof10110764

**Published:** 2024-11-02

**Authors:** Sepideh Fekrikohan, Bahram Sharifnabi, Mohammad Javan-Nikkhah, Stefania Pollastro, Francesco Faretra, Rita Milvia De Miccolis Angelini

**Affiliations:** 1 Department of Plant Protection, College of Agriculture, Isfahan University of Technology, Isfahan 8415683111, Iran; fekrikohan2017@gmail.com; 2 Department of Plant Protection, College of Agriculture and Natural Resources, University of Tehran, Tehran 1417466191, Iran; jnikkhah@ut.ac.ir; 3 Department of Soil, Plant and Food Sciences, University of Bari Aldo Moro, Bari 70126, Italy; francesco.faretra@uniba.it (F.F.); ritamilvia.demiccolisangelini@uniba.it (R.M.D.M.A.)

**Keywords:** grey mould, molecular characterization, rose, strawberry, raspberry

## Abstract

Grey mould disease, caused by various *Botrytis* species, poses a significant threat to important plants worldwide. This study aimed to characterize *Botrytis* populations on strawberry and roses, economically relevant host plants, and raspberry, used as a representative of wild plants, in Iran. A total of 389 isolates were collected and analyzed based on morphological features and haplotyping using molecular markers, transposable elements (*Boty* and *Flipper*), and fungicide response. Moreover, 60 isolates were used for phylogenetic analysis based on the *rpb2* gene, and 16 selected isolates from each clade were further characterized using the *g3pdh*, *hsp60*, and *nep2* genes. The results revealed the presence of three distinct species, *Botrytis cinerea*, *Botrytis sinoviticola*, and *Botrytis prunorum*, among the sampled isolates. Additionally, this study reports for the first time the presence of *B. sinoviticola* on strawberry and isolates belonging to *B. cinerea* group S in Iran. These findings provide insights into the diversity and composition of *Botrytis* populations on Iranian host plants.

## 1. Introduction

In recent decades, Iran has emerged as a significant global producer of various crops susceptible to *Botrytis* species, as reported by FAOSTAT [1]. The cultivation of high-value crops, such as strawberry (*Fragaria ananassa* Duchesne) and roses (several species of the genus *Rosa* L.), has increased, particularly in greenhouse environments, which often provide favourable conditions for *Botrytis* growth. *Botrytis* species can cause various diseases, such as blossom blight, leaf blight, and onion neck rot, but the most relevant is grey mould [2]. Grey mould disease, induced by *Botrytis cinerea*, results in heavy yield losses, causing severe economic losses all over the world, especially during periods of high humidity before harvest [3]. The ability of *Botrytis* species to cause latent infections in host tissues also poses significant challenges during transport and storage, making them formidable postharvest pathogens.

The genus *Botrytis* belongs to the *Leotiomycetes* class, *Helotiales* order, and *Sclerotiniaceae* family within the *Ascomycota* phylum [4], comprising approximately 38 species, either polyphagous or specialized, that collectively infect over 1400 plant species [5,6]. Notably, *Botrytis*, as a saprophyte, can continue to grow and survive on host plants even after their decay, posing a considerable challenge in disease management [7].

Traditionally, species identification relied on morphological and cultural characteristics [8]. However, morphological similarities hampered identification, and difficulties were overcome by the development of molecular methods based on DNA sequencing [9]. Molecular techniques provide valuable insights into pathogen detection, species identification, and genetic variability within *Botrytis* species and are helpful in early diagnosis, early-stage treatments, and the adoption of suitable antifungal control measures [10].

*B. cinerea*, the most prominent species within the genus, is in second place in the list of the top 10 fungal plant pathogens in the world based on their scientific and economic importance, preceded only by *Magnaporthe oryzae* [11].

*B. cinerea* shows considerable genetic variation, which has been well documented in the literature from the early studies of the fungus [9], and which makes it a high-risk pathogen for the development of fungicide resistance (FRAC; http://www.frac.info (accessed on 20 July 2024)). Two transposable elements (TEs), the retrotransposon *Boty* and the DNA transposon *Flipper*, have been identified in *B. cinerea* and used to define two sibling sympatric species or biotypes, named *transposa* and *vacuma*, based on the presence or absence of the two TEs [12]. Significant differences in the frequency and distribution of the two transposons among *Botrytis* isolates collected from different host plants and/or geographic regions have been reported [13,14]. Moreover, a polymorphic exon/intron structure in the mitochondrial *cytb* gene was reported in *B. cinerea*, with isolates possessing or lacking a group I intron interrupting the coding sequence of the gene [15].

Several molecular markers have been used for genetic identification and phylogenetic relationships within *Botrytis* species. Phylogenetic analysis has divided the genus *Botrytis* into two clades based on the *Bc-hch* gene sequence, with *B. cinerea* falling under the clade of species (*Botrytis* clade 1) that infects a wide host range of dicotyledonous plants and is clearly distinguished from clade 2, which contains *Botrytis* species with a restricted host range in both monocotyledonous and dicotyledonous plants [16]. Three housekeeping genes, glyceraldehyde-3-phosphate dehydrogenase (*g3pdh*), heat shock protein 60 (*hsp60*), and subunit II RNA polymerase (*rpb2*), and the necrosis- and ethylene-inducing protein (*nep1* and *nep2*) genes, have been used to corroborate the identification of *Botrytis* species [17,18,19,20]. Additionally, the sequencing of the multidrug resistance regulator 1 (*mrr1*) gene and several other genes distinguished a subgroup of isolates within grey mould populations, named *B. cinerea* group S, that was found to be predominant on strawberry in Germany [21]. These findings further highlight the need for accurate species identification and understanding intraspecific variability.

Numerous studies have examined genetic polymorphisms in *Botrytis* isolates collected from diverse host plants and growing systems worldwide [4].

The commercial significance of strawberry and roses in Iran and the potential transfer of isolates between different host plants prompted us to conduct the morphological and molecular characterization of *Botrytis* isolates from these crops and wild *Rosaceae* raspberry plants, in different provinces, to shed light on the diversity and distribution of *Botrytis* populations in Iran.

## 2. Materials and Methods

### 2.1. Fungal Isolates

The collection of *Botrytis* isolates sampled from various host plants exhibiting typical grey mould symptoms in open fields or in greenhouses is detailed in Table 1. Isolates were obtained by extracting small fragments of developed mycelia and/or conidia, or from surface-sterilized petals, leaves, or fruits without visible fungal material, which were then cultured on PDA medium (infusion from 200 g peeled and sliced potatoes kept for 1 h at 60 °C, 20 g dextrose, adjusted to pH 6.5, 20 g LLG-European bacteriological agar, per litre of distilled water). The cultures were maintained at 21 ± 1 °C in the dark for 7 days. Subsequently, fungal isolates were purified using the single-spore method and identified based on their morphological macroscopic and microscopic traits (e.g., colony morphology, conidia shape and size, sclerotia shape, and size and distribution pattern on Petri plates). Regarding storage, the isolates were maintained on paper slants at −20 °C for short-term storage or in 10% glycerol at −80 °C for long-term storage.

### 2.2. Genetic Analysis and Fungicide Resistance

The single-spore isolates were cultured on malt extract agar (MEA; 20 g malt extract Oxoid, 20 g agar, per litre) at 21 ± 1 °C in darkness for 2–3 days. Subsequently, five small (2–4 mm) mycelial agar plugs from each isolate were singularly transferred onto 90 mm MEA plates overlaid with sterile cellophane films. The cultures were then incubated at 21 ± 1 °C for an additional two days to obtain fresh mycelium. Genomic DNA extraction was carried out from freeze-dried mycelium using the CTAB method as described by De Miccolis Angelini et al. [22]. The quality and concentration of the extracted DNA were determined using a Nanodrop 2000 spectrophotometer (Thermo Fisher Scientific Inc., Wilmington, DE, USA), and it was stored at −20 °C until use.

All isolates underwent molecular analysis: (i) TE profiles were determined using PCR primers specific to *Boty* and *Flipper* [23]; (ii) *cytb* gene structure was assessed according to De Miccolis Angelini et al. [15] and Habib et al. [24]; (iii) strains belonging to group I or group II of the *Botrytis* genus were distinguished by *Bc-hch* RFLP analysis [25]; (iv) *B. cinerea* group S strains were identified by the *mrr1* PCR assay [21]. In detail, the PCR mixtures (25 μL) consisted of 1× Green GoTaq Flexi Buffer (Mg^2+^ free), 2 mM MgCl_2_, 75 μM of each dNTP nucleotide, 0.5 μM of each primer, 0.75 U of GoTaq DNA polymerase (all PCR reagents were from Promega Corp., Madison, WI, USA), and 50 ng of genomic DNA. The primer pairs used are listed in Table 2. A control with no template was always run. *HhaI* restriction enzyme (New England Biolabs Ltd., Hitchin, UK) was employed for *Bc-hch* RFLP analysis in a 10 µL mixture consisting of 1× CutSmart™ Buffer, 2 U of enzyme, and 4 µL of PCR product, and the digestion reaction was carried out at 37 °C for 30 min. The results were visualized after 110 min of electrophoresis run on a 1.5% agarose gel at 110 V using SYBR Safe DNA gel stain (Thermo Fisher Scientific, Waltham, MA, USA).

Sensitivity tests were conducted for six selected fungicides, namely, the SDHIs boscalid and isofetamid, the class III sterol biosynthesis inhibitor (SBI-III) fenhexamid, the phenylpyrrole fludioxonil, the anilinopyrimidine pyrimethanil, and the Quinone outside Inhibitor (QoI) trifloxystrobin, due to their frequent usage and/or having a common mode of action with the fungicides most frequently used against *B. cinerea* in recent years in Iran. Isolates were individually tested for their response to each fungicide by colony growth tests. In brief, mycelial plugs (2–4 mm) from the margins of actively growing colonies were placed upside-down on fungicide-amended or unamended media. The composition of the media and the discriminating doses of the fungicides were as previously described by De Miccolis Angelini et al. [26]. After incubation at 21 ± 1 °C in darkness for 2–5 days, isolates showing colony growth were considered to be resistant, while isolates whose growth was inhibited were considered to be sensitive to the tested fungicide. Reference *B. cinerea* strains already known for their sensitivity or resistance to each fungicide were used as controls in each test.

The haplotyping categorization integrated data from both genetic analysis and fungicide response. One isolate from each haplotype was then selected for further genetic analysis.

### 2.3. Phylogenetic Analysis

Phylogenetic analysis was conducted using the *rpb2*, *g3pdh*, *hsp60*, and *nep2* gene sequences. The PCR was performed in a 20 μL reaction mixture consisting of 1× Phusion™ HF Buffer, 0.2 mM dNTPs, 0.5 μM of each primer, 0.4 U of Phusion™ high-fidelity DNA polymerase (all PCR reagents were from Thermo Fisher Scientific), and 25 ng of template DNA. The reaction was carried out in a MyCyclerTM thermal cycler (Bio-Rad Laboratories, Hercules, CA, USA) programmed for initial denaturation at 98 °C for 30 s, followed by 35 cycles of denaturation at 98 °C for 10 s, annealing at 51–64 °C for 10 s, and extension and final extension at 72 °C for 0.5 and 8 min, respectively. The primer sequences and annealing temperatures related to all the markers used in the analysis are shown in Table 2. It should be noticed that the NEP2forE/NEP2revE primer pair was used in addition to the more commonly used NEP2(−200)for/NEP2(+1147)rev primer pair to obtain high-quality *nep2* sequences from *B. prunorum* isolates, according to Staats et al. [17].

PCR products were directly sequenced in both forward and reverse directions, using the same primers as for PCR, from an external service (Genewiz from Azenta Life Sciences, Leipzig, Germany). DNA sequence analysis was carried out using the Lasergene software package (v. 15.0.1; DNASTAR Inc., Madison, WI, USA). In detail, the nucleotide sequences for each gene were aligned using ClustalW and default settings in MegAlign Pro software (Lasergene v. 15.0.1; DNASTAR Inc.). After filtering out the poorly aligned regions, alignments for *rpb2*, *g3pdh*, and *hsp60* were concatenated and used to reconstruct the maximum likelihood (ML) tree with 1000 bootstrap replicates with ASTRAL-II, according to Eyvazi et al. [27]. The ML tree for *rpb2* and *nep2* was inferred using MEGA7 [28] with similar parameters. *Sclerotinia sclerotiorum* strain 484 was used as the outgroup. The code of each sequence used for building phylogenetic trees is listed in Supplementary Table S1.

### 2.4. Pathogenicity Assay

The pathogenicity of the *B. sinoviticola* strain, three strains of *B. prunorum*, and three strains of *B. cinerea* (Supplementary Table S2) was evaluated by artificial inoculation on healthy organic strawberry fruits (cv ‘Candonga’) and cucumber cotyledons (cv ‘Mezzo Lungo di Polignano’), which were previously decontaminated by immersion in 2% sodium hypochlorite for 1 min, washed twice with sterilized distilled water, and dried at room temperature. Strawberry fruits were inoculated with conidial suspensions prepared using sterile distilled water containing 0.01% Tween 20 from 7-day-old cultures grown on PDA. Conidial suspensions were then filtered through a layer of Miracloth (Calbiochem, San Diego, CA, USA) to remove mycelium fragments and adjusted to 1 × 10^5^ conidia mL^−1^ using a haemocytometer. Each fruit was punctured with a sterile needle after being inoculated with 20 µL of conidial suspension. Cucumber cotyledons were wounded and inoculated with a mycelium plug (2 to 4 mm in diameter) excised from the margin of actively growing cultures on MEA. Fruit inoculated with sterile distilled water and cotyledons inoculated with plugs of sterile MEA were used as a control. After inoculation, fruit and cotyledons were incubated in a moist chamber at 21 ± 1 °C in darkness. Starting from 2 days after inoculation, rotting around the inoculation point was recorded according to an empirical scale with seven classes of severity (0 = absence of infections, 1 = a lesion < 1 mm of rotted area (r.a.); 2 = 1–3 mm of r.a.; 3 = 4–5 mm r.a.; 4 = 6–7 mm r.a.; 5 = up to 50% r.a.; 6 = 51–75% r.a.; 7 = 76–100% r.a.). Data from five replicated fruits/cotyledons were used to calculate the mean disease severity. The assay was repeated twice. All data were analyzed by analysis of variance followed by Tukey’s honestly significant different test using CoStat software version 6.451 (CoHort Software, Monterey, CA, USA) at the significance level *p* = 0.05.

## 3. Results

### 3.1. Fungal Isolates

**Figure 1:**
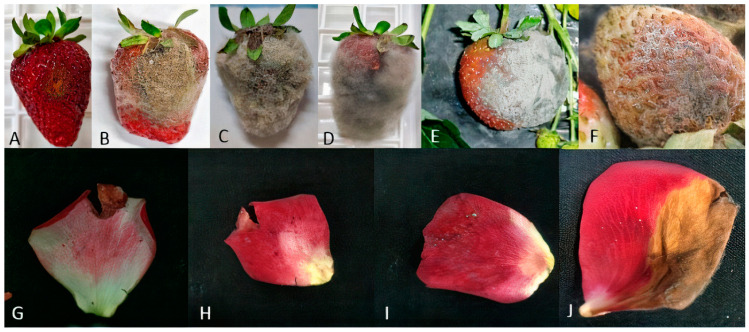
Grey mould symptoms on strawberry fruits (**A**–**F**) and rose petals (**G**–**J**).

The typical symptoms associated with grey mould included spreading lesions on fruits or petal decay, with or without visible conidia, and/or mycelium on the surface. Some examples of symptomatic tissues are reported in Figure 1. In this study, both symptomatic and asymptomatic materials were utilized, showing the presence of latent infections of *B. cinerea* in the analyzed samples. Following an incubation period on PDA medium of 4–7 days at 21 °C in darkness, fungal mycelium and sporulation appeared and were utilized for single-spore purification to obtain a collection of *Botrytis* isolates. A total of 389 *Botrytis* isolates, first identified on the grounds of morphological characteristics, were obtained. The colony morphology of isolates was classified into three main categories (mycelial, conidial, and sclerotial) and eleven different morphotypes, as detailed in Supplementary Table S3. The isolates encompassed all categories, with the conidial one being dominant, but morphology could vary among subcultures from the same isolate. However, based on morphological features, all isolates appeared to be consistent with *B. cinerea*.

### 3.2. Grouping of Isolates Based on Genetic Analyses and Fungicide Resistance

**Figure 2:**
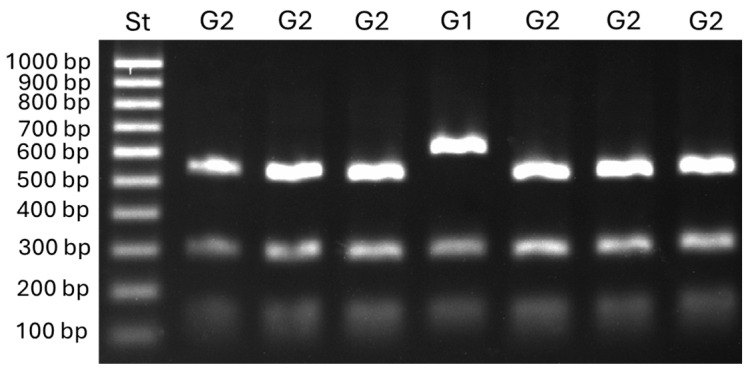
Restriction profiles of *Botrytis* group 1 (G1) and group 2 (G2) isolates obtained with the *Bc-hch* PCR-RPLP assay according to Fournier et al. [26] (PCR amplification with the primer pair 262/520L followed by digestion with the restriction enzyme *HhaI*).

DNA amplification for the detection of the TEs *Boty* and *Flipper* revealed, in the analyzed isolates, all four transposon combinations, namely, *transposa* (Boty^+^Flipper^+^; 65%), Boty (Boty^+^Flipper^−^; 25%), Flipper (Boty^−^Flipper^+^; 7%), and *vacuma* (Boty^−^Flipper^−^; 2%). Further analysis involving the restriction of *Bc-hch* amplicons indicated that one isolate belonged to group I and the remaining ones belonged to group II (Figure 2). Both intron-possessing (T1; 42%) and intron-lacking (T2; 58%) variants of the *cytb* gene were detected. Additionally, 24 isolates (6.1%) from different host plants belonged to *B. cinerea* group S.

The fungicide response of 345 out of the 389 isolates was categorized as sensitive (S) or resistant (R) to each fungicide (Supplementary Table S3). In addition, isolates showing low (LR) to high (HR) levels of resistance were distinguished for fenhexamid and fludioxonil. As a result, based on their response profiles for the assayed fungicides, the isolates were classified into a total of 44 different groups, with most of the isolates resistant to at least one class of fungicide (95%).

Overall, 60 haplotypes were identified among the analyzed *Botrytis* isolates based on data from molecular data and fungicide responses (Table 3).

### 3.3. Phylogenetic Analysis

**Figure 3:**
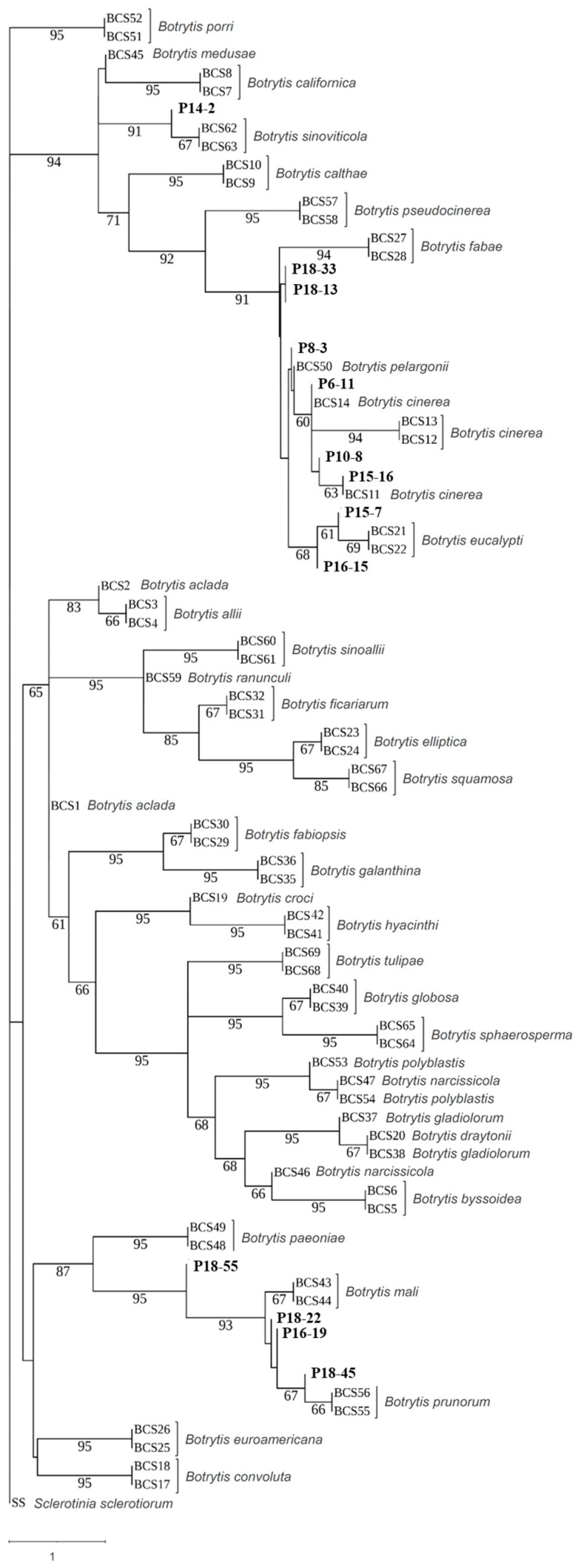
Multi-gene phylogenetic tree based on concatenated *rpb2*, *g3pdh*, and *hsp60* gene sequences prepared by using Asteral software. Iranian strains from the current study are in bold. The tree is drawn to scale, with branch lengths measured by the number of substitutions per site. Bootstrap values > 60 based on 1000 replicates are shown.

Preliminary, the *rpb2* gene sequences of 60 selected isolates representative of different haplotypes were analyzed. The two *B. cinerea* group S isolates, P12-28 and P16-24, and the isolate P15-2 did not produce sequences of good quality with *rpb2* primers and were the excluded from subsequent analysis. Afterward, 16 isolates were selected based on macroscopic and microscopic morphology and the *rpb2* gene sequences and subjected to molecular species identification based on the concatenated sequences of the *rpb2*, *g3pdh*, *hsp60*, and *nep2* genes. Molecular analysis, with few exceptions, confirmed the results of the morphological observations. The initial analysis based on the *rpb2* gene (Supplementary Figure S1A) grouped 38 of the 57 examined isolates in the previously described *Botrytis* clade 1 [16,17,20], including *B. cinerea* and the closely related species *Botrytis fabae*, *Botrytis pseudocinerea*, *B. sinoviticola*, *Botrytis californica*, *Botryotinia calthae* (syn. *Botrytis calthae*), and *Botrytis medusae* used in our analysis as references. Among them, the P14-2 strain was closely related to *B. pseudocinerea* and *B. sinoviticola*, while all the other isolates grouped close to *B. cinerea*. The remaining 19 isolates clustered together in a separated clade with the reference sequences of *B. prunorum*. Sequence analysis of *nep2* (Supplementary Figure S1B) corroborated these results. It should be mentioned that the second group of *nep2* primers (Table 2) produced more qualified amplicons and was able to place *B. prunorum* isolates into separated subgroups. The phylogenetic analysis using the combined *rpb2*, *g3pdh*, and *hsp60* sequences on the selected 16 isolates (Figure 3) consistently placed the strain P14-2 with *B. sinoviticola* in one subclade, forming a single lineage within *Botrytis* clade 1, and confirmed the molecular identification of 5 isolates as *B. prunorum* and 10 isolates as *B. cinerea* (Table 4). The *nep2* data were not included in the construction of the combined phylogenetic tree due to the lack of information related to all the reference sequences used for this gene in the NCBI database. On the other hand, our results confirmed the insufficiency of single marker genes in species identification.

### 3.4. Morphological Analysis and Fungicide Resistance and Pathogenicity

**Figure 4:**
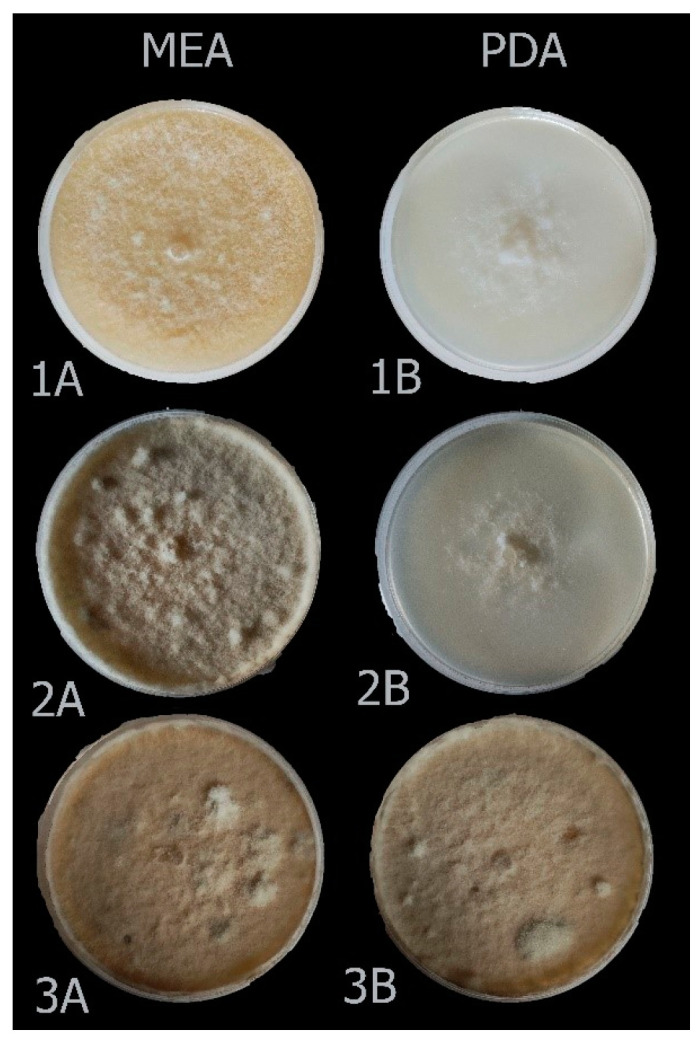
Colony morphology of *Botrytis sinoviticola* strain P14-2 on MEA (**A**) and PDA (**B**) after 7 (1), 14 (2), and 21 (3) days.

**Figure 5:**
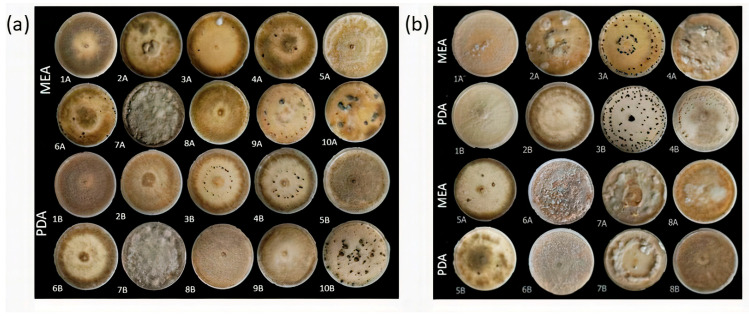
Various colony morphologies of *Botrytis cinerea* (**a**) and *B. prunorum* (**b**) on MEA and PDA after 21 days of incubation. Among *B. cinerea* strains, 1—P13-5; 2—P11-4; 3—P6-9; 4—P8-11; 5—P2-2; 6—P3-3; 7—P7-4; 8—P18-29; 9—P18-6; and 10—P7-3; among *B. prunorum* strains, 1—P11-14; 2—P10-3; 3—P11-10; 4—P16-17; 5—P16-19; 6—P8-9; 7—P18-38; and 8—P18-22.

**Figure 6:**
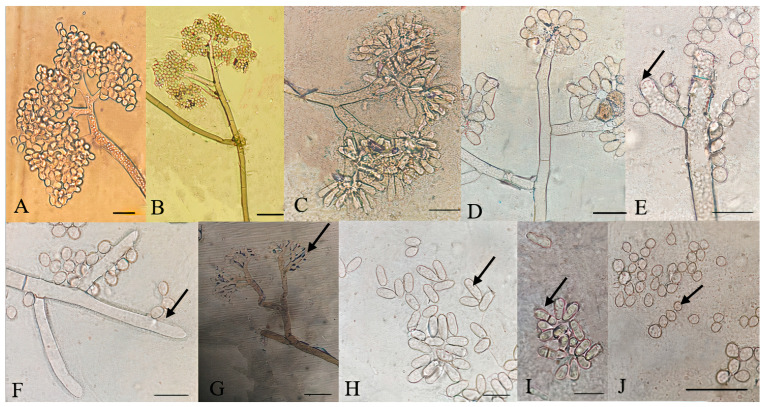
Microscopic characteristics of *Botrytis cinerea* isolates. (**A**–**D**) Conidia attached to conidiophore, (**E**–**G**) conidiophore terminal, (**H**) elongated conidia, (**I**) septate conidia, (**J**) microconidia. Bar = 20 μm. The strains shown are (**A**) P13-5; (**B**) P8-11; (**C**) P7-4; (**D**) P18-6; (**E**) and (**F**) P7-3; (**G**) P3-3; (**H**) P7-4; (**I**) P2-2; and (**J**) P7-3.

**Figure 7:**
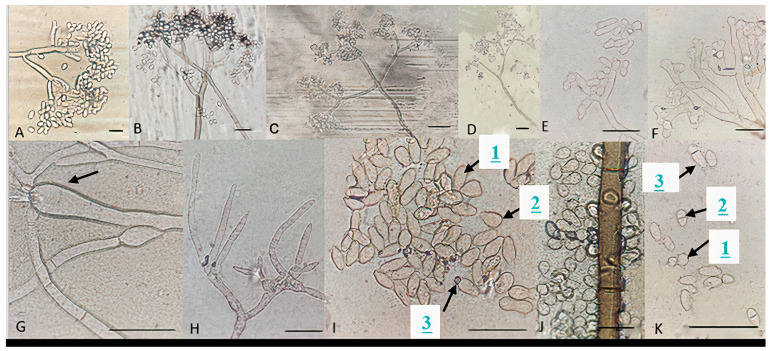
Microscopic characteristics of *Botrytis prunorum*. (**A**–**D**) Conidia attached to conidiophore and a normal (**E**), swollen (**F**), and thin (**H**) conidiophore terminal. (**G**) Conidiophore swelling, elongated (1I), and pyriform conidia (2I), conidium with hilum (3I), (**J**) common conidia, and round (1K), small (2K), and large (3K) septate conidia. Bar = 20 μm. The strains shown are (**A**) P11-14; (**B**) P16-17; (**C**) P18-38; (**D**) P8-9; (**E**) P10-3; (**F**) P11-10; (**G**) P18-22; (**H**) P16-19; (**I**) P10-3; (**J**) P8-9; and (**K**) P10-3.

**Figure 8:**
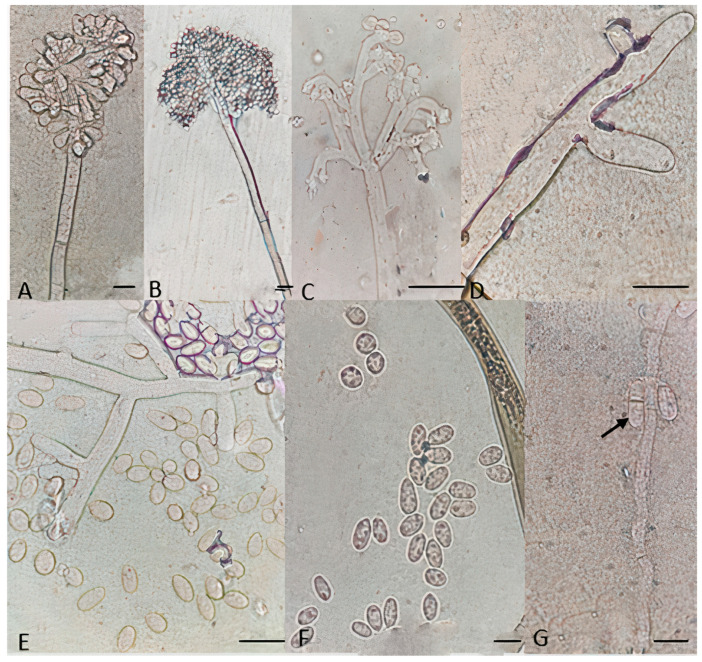
Microscopic characteristics of *Botrytis sinoviticola* strain P14-2. (**A**,**B**) Conidia attached to conidiophore, (**C**,**D**) conidiophore terminal, (**E**,**F**) unicellular conidia, and (**G**) septate conidia. Bar = 20 μm.

For all identified species within the analyzed *Botrytis* populations, conidia were ovate, ellipsoidal, pyriform, globose, flat in one part, usually unicellular but occasionally septate, and with or without hilum. Their sizes exhibited broad variation, in the range of 3–19 × 3–9 μm (*n* = 50). Macroscopic and microscopic morphological features were not useful in distinguishing the *Botrytis* species investigated (Figure 4, Figure 5, Figure 6, Figure 7 and Figure 8). The *B. sinoviticola* strain from this study yielded colonies without sclerotia with a creamy to grey colour on MEA and whitish to grey on PDA (Figure 4). The colony morphology of *B. cinerea* and *B. prunorum* strains was somehow affected by the medium (PDA and MEA). For example, *B. cinerea* strain 6 (P3-3) and *B. prunorum* strain 2 (P10-3) produced sclerotia on MEA but not on PDA, while *B. cinerea* strain 5 (P2-2) and *B. prunorum* strain 4 (P16-17) produced sclerotia just on PDA. Strain 3 from both species (P6-9 and P11-10) produced more sclerotia on PDA, while strain 1 of *B. cinerea* (P13-5) and strain 8 of *B. prunorum* (P18-22) had more conidia on PDA.

Particular microscopic features, like swelling at the junction of conidiophore in some *B. prunorum* strains (Figure 7G), and a little curviness at the end of conidiophore in *B. sinoviticola* strains (Figure 8C), were observed. It should be noticed that the features mentioned were not specific to a single species, since they could sometimes also be observed in strains representative of other species.

**Figure 9:**
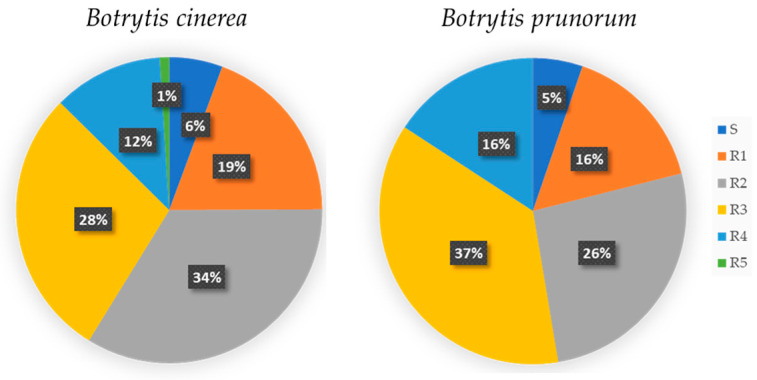
Distribution pattern of fungicide resistance profiles among *Botrytis cinerea* and *Botrytis prunorum* strains, grouped in the following categories: sensitive to all used fungicides (S), and resistant to one (R1), two (R2), three (R3), four (R4), and five (R5) classes of fungicides.

With regard to fungicide response, the *B. sinoviticola* strain showed double resistance to fludioxonil and SDHIs and sensitivity to fenhexamid, anilinopyrimidines, and QoI fungicides (haplotype H17). The occurrence of sensitivity (S) or single (R1), double (R2), or multiple (R3–R5) resistance to the fungicides tested among *B. cinerea* and *B. prunorum* strains is summarized in Figure 9, showing similar proportions in the two species.

A pathogenicity test was carried out for Iranian *B. sinoviticola*, firstly identified in this study, compared with strains of *B. cinerea* and *B. prunorum* on strawberry fruits and cucumber cotyledons. In two replicated experiments, *B. sinoviticola* caused lesions starting from 3 DAI on strawberry and 4 DAI on cucumber and reaching the maximum disease severity after 6 DAI and 7 DAI on the two hosts, respectively. No significant difference was observed among strains of the three *Botrytis* species (Supplementary Table S2).

## 4. Discussion

*Botrytis* species include plant pathogens that affect a large number of crops [4]. This study focused on the *Botrytis* species associated with two important economic crops, strawberry and rose, and a wild plant, raspberry, in Iran. Since different *Botrytis* species can convive on a single host plant showing grey mould symptoms [2,29], we aimed to investigate the composition of *Botrytis* populations to improve integrated disease management. The diversity observed in colony growth and colour, the number and pattern of sclerotia formations, the sequences of the genes *Bc-hch* and *mrr1*, the mitochondrial *cytb* gene, and the presence of the TEs *Boty* and *Flipper* [30], along with the response to different fungicides, was initially used for grouping the sampled *Botrytis* isolates. This led to the identification of 60 different haplotypes in fungal populations and to the selection of one isolate per haplotype for submission for phylogenetic analysis.

Species identification and phylogenetic analysis was achieved with multilocus analysis by using the gene sequences of *g3pdh*, *hsp60*, *rpb2*, and *nep2* [16,17]. Both morphological and molecular studies showed the prevalent presence of *B. cinerea*, including isolates of group S. Isolates of *B. prunorum* and *B. sinoviticola* were also detected. It should be mentioned that no species-specific or haplotype-specific macroscopic or microscopic features could be recorded, and some features, like occasional septate conidia, were detected in different *Botrytis* species, in agreement with Mirzaei et al. [31]. These results confirm the broad intraspecific variation that is well known in *B. cinerea* and corroborate previous observations showing broad morphological variation in other *Botrytis* species [9]. Differences in colony morphologies in *B. cinerea* and *B. prunorum* grown on various culture media were like those reported in other studies [32]. We also observed that the morphology of monoconidial isolates was not always stable following repeated subculture and seriously questioned the reliability of morphological descriptions of cultured isolates in species identification [9]. It should be noticed that our morphological observations were always conducted on fungal colonies grown under dark conditions and that light exposure could affect mycelial growth, conidiation, sclerotial development, and trophic responses in *Botrytis* [33].

Consistent with previous findings on different hosts, *B. cinerea sensu stricto* was identified as the main pathogen and the one most frequently associated with grey mould on various plants [34,35], although, in some instances it may be partially replaced by *B. pseudocinerea* during the growing season [2]. However, *B. pseudocinerea* was not found among the sampled isolates in this study.

As expected, *B. cinerea* isolates were all recognized as belonging to *Botrytis* group II based on the *Bc-hch* RFLP test, showed considerable morphological and genetic variation, with various profiles of response to fungicides and contents in TEs, including *transposa* (Boty^+^Flipper^+^), Boty^+^Flipper^−^, Boty^−^Flipper^+^, and *vacuma* (Boty^−^Flipper^−^) isolates, as well as both structural variants of the *cytb* gene: possessing (T1) or not possessing (T2) the intron. Isolates belonging to *Botrytis* group S were identified based on the *mrr1* PCR test, and this is the first report of this group in Iran. *Botrytis* group S was initially identified on strawberry in Germany [21], exhibiting host specificity at that time. However, subsequent findings considerably expanded its host range to various other plants across different countries [30,34,36,37], highlighting a lack of host specificity.

Several studies define *B. cinerea* as a species complex in which *B. cinerea sensu strictu* exists within a species complex, including *B. pseudocinerea* and new cryptic species that can live in sympatry in the same host. Although the *Botrytis* species in the complex show morphological similarity, they may differ with respect to ecology, host preferences, aggressiveness, and sensitivity to fungicides [12,13,19,20,25,38].

*B. prunorum*, one of the cryptic species in the *B. cinerea* species complex, shares phylogenetic similarities with *B. cinerea* [8]. The species has been reported in various countries and on various taxonomically unrelated plant species. It was first detected and characterized from Japanese plum [32], table grape [39], and kiwifruit in Chile [40], then it was also detected from dry pea, lentil, and chickpea in Montana, USA [41], strawberry in Norway [36], greenhouse-grown tomato in Turkey [42], and vineyards in Spain [41]. In this study, it was found in multiple provinces in Iran on strawberry (in all provinces, except Gilan), raspberry (in Mazandaran province), and roses (in Alborz and Gilan provinces). At first, it was distinguished from *B. cinerea* because it yielded a white-to-yellow colony on PDA, shorter and more compressed conidiophores at the base, smaller conidia and sclerotia, and less sporulation [32,39]. However, our study, in agreement with Nabizadeh et al. [2] (2022), revealed many overlapping morphological features among *B. cinerea* and *B. prunorum*. Molecular analysis using *rpb2*, *nep2*, and the combined *rpb2*, *g3pdh*, and *hsp60* gene sequences clustered Iranian isolates with the reference sequences of *B. prunorum*. The *B. prunorum* isolates characterized in this study all belonged to *Botrytis* group II and included different haplotypes showing various TEs and fungicide resistance profiles and both intron variants of the *cytb* gene.

Isolates belonging to Botrytis group I based on the *Bc-hch* gene were identified as *B. sinoviticola* based on molecular and morphological analyses and according to the holotype firstly described by Zhou et al. [19]. Isolates were characterized by the presence of the only *Flipper* transposon (Boty^−^Flipper^+^) in their genomic DNA and the presence of the intron in the mitochondrial *cytb* gene and showed resistance to fludioxonil and SDHIs and normal sensitivity to fenhexamid and the other classes of fungicides tested. Pathogenicity on strawberry fruits and cucumber cotyledons was demonstrated in in vitro assays. *Botrytis* group I isolates were initially designated as a single new species, *B. pseudocinerea* [43], but additional species have been subsequently characterized, including *B. calthae* [44], *B. californica* [38], and *B. sinoviticola* [19]. *B. sinoviticola* was firstly described as another cryptic species living in sympatry with *B. cinerea* on table grapes in China [19], and it was found on strawberry leaves collected in an open field during spring in Mazandaran province in the current study. The species was distinguished from others based on the formation of numerous round and small sclerotia on PDA and villiform appendages on the conidial surface observed under an electron microscope [19]. Previous reports from Iran documented its presence on pomegranate from Fars province [45] and grapes and apple from Kurdistan province [2]. Our isolates showed a white-to-creamy colony and other morphological features like those mentioned in the first description of the species [19]. Mycelial colonies with no sclerotia and oval-to-ovoid conidia were also like those previously reported by Nabizadeh et al. [2]. Multilocus analysis using *rpb2*, *g3pdh*, and *hsp60* sequences clearly distinguished *B. sinoviticola* Iranian isolates from others, originating a single lineage within *Botrytis* clade 1 and forming a well-supported clade together with the reference *B. sinoviticola* strains.

This study represents the first report of *B cinerea* group S and *B. sinoviticola* from strawberry in Iran. It should be noted that the highest proportion of *B. cinerea* group S isolates were related to P12 and P18 populations (both from Kurdistan province), respectively. Previously, reports of *B. sinoviticola* had been limited to China and Iran, from pomegranate in Fars province and apple and vine in West Kurdistan province; this study extended the host range and distribution of this species to the Mazandaran province in Iran.

In conclusion, our results highlighted several key points: (1) *B. cinerea* group S isolates are not host-specific, as we could identify them on both strawberry and roses in different provinces; (2) while some species-specific morphological features, such as occasionally septate conidia or the swelling of the conidiophore may be observed, especially when studying numerous isolates, reliable differentiation without molecular studies is challenging due to the broad variability and instability of morphological traits [2]; (3) despite rigorous efforts towards species distinction through a combined morphological and molecular approach, certain species might be not separated, likely due to the coevolution of genes used in multilocus analysis; (4) contrasting with previous reports by Johnston et al. [30], we could not find any relationship between the content of TEs and *Botrytis* species, since we detected isolates of the B^−^F^−^ type among *Botrytis* group II, and the only *Botrytis* group I isolate tested was of the B^+^F^−^ type.

These findings contribute to the understanding of *Botrytis* species diversity, emphasizing the importance of molecular techniques in accurate species identification and providing valuable insights into the genetic and ecological dynamics of *Botrytis* populations on cultivated and wild host plants in Iran. This information is particularly useful in improving the management of diseases on economically important hosts.

## Figures and Tables

**Table 1:** Origin of the *Botrytis* isolates used in this study.

Host	Population	Origin of Transplant	Province	City	Sampling Time	Growing System	Cultivar
Raspberry	P1	-	Gilan	Kiashahr	Spring 2021	Jungle	Wild plant
	P2	-	Mazandaran	Babol	Spring 2021	Jungle	Wild plant
Roses	P3	-	Alborz	Karaj	Autumn 2022	Garden	Floribundas
	P4	-	Markazi	Arack	Autumn 2022	Greenhouse	Hybrid teas
	P5	-	Isfahan	Isfahan	Autumn 2022	Greenhouse	Hybrid teas
	P6	-	Hamedan	Hamedan	Autumn 2022	Greenhouse	Hybrid teas
	P7	-	Isfahan	Kashan	Autumn 2022	Greenhouse	Hybrid teas
	P8	-	Gilan	Kiashahr	Autumn 2022	Greenhouse	Hybrid teas
	P9	-	Mazandaran	Noor	Autumn 2022	Garden	Floribundas
	P10	-	Fars	Shiraz	Autumn 2022	Greenhouse	Hybrid teas
	P17	-	Khuzestan	Dezful	Autumn 2022	Greenhouse	Hybrid teas
Strawberry	P11	Hashtgerd	Alborz	Hashtgerd	Spring 2021	Greenhouse	Camarosa
	P12	Sanandaj	Kurdistan	Chenareh	Spring 2021	Open field	Camarosa
	P13	Hashtgerd	Gilan	Lahijan	Spring 2021	Greenhouse	Camarosa
	P14	Hashtgerd	Mazandaran	Behnamir	Spring 2021	Open field	Sabrina
	P15	Hashtgerd	Kerman	Jiroft	Winter 2022	Greenhouse	Camarosa
	P16	Orumiyeh	Isfahan	Isfahan	Spring 2021	Greenhouse	Sabrina/Albion
	P18	Sanandaj	Kurdistan	Kamyaran	Spring 2021	Greenhouse	Camarosa

- = unknown.

**Table 2:** PCR primer pairs used in this study.

Gene	Primer	Sequence (5′-3′)	Annealing Temperature	Amplicon Size (bp)	Reference
***Flipper***	F300 F1550	GCACAAAACCTACAGAAGA ATTCGTTTCTTGGACTGTA	60 °C	1250	[23]
***Boty***	B1830 B2800	ATAAAGAAGCAACCGGATGG AGTCTATCGGGTCCATCCTT	60 °C	970	
***cytb***	Cytb.139 Cytb.872	ACCGAATGGTGGGATCAATA ATGCCCTCAAAAGGGGATAG	55 °C	734	[15]
***Bc-hch***	262 520L	AAGCCCTTCGATGTCTTGGA ACGGATTCCGAACTAAGTAA	55 °C	1171	[25]
***mrr1***	Mrr1-spez-F Mrr1-spez-R	TATCGGTCTTGCAGTCCGC TTCCGTACCCCGATCTTCGGAA	51 °C	144–165	[21]
***rpb2***	RPB2for+ RPB2rev+	GATGATCGTGATCATTTCGG CCCATAGCTTGCTTACCCAT	51 °C	1184	[16]
***hsp60***	HSP60for+ HSP60rev+	CAACAATTGAGATTTGCCCACAAG GATGGATCCAGTGGTACCGAGCAT	51 °C	981	
***g3pdh***	G3PDHfor+ G3PDHrev+	ATTGACATCGTCGCTGTCAACGA ACCCCACTCGTTGTCGTACCA	64 °C	876	
***nep2***	NEP2(−200)for NEP2(+1147)rev	GAACTTTGAATAGTGGGCAGTTGGG GAGTTTCAGGTATATTCGTTTGGTGGA	51 °C	1347	[17]
	NEP2forE NEP2revE	gtgactgtaaaacgacggccagtTCATCATGGTTGCCTTCTCAAGAT gtgaccaggaaacagctatgaccAAGTAGCAGCTGCAAGATTGTTTG	51 °C	845	

**Table 3:** Description of the haplotypes detected among the collected isolates.

Selected Isolate	Haplotype	Fungicide Response ^a^	TEs ^b^	*mrr1* ^c^	*Bc-hch* ^d^	*cytb* ^e^
P18-13	H1	SDHI^R^ AP^R^ QoI^S^ Fen^HR^ FLu^HR^	B^+^F^+^	W	G2	T1
P18-6	H2	SDHI^R^ AP^S^ QoI^S^ Fen^HR^ FLu^LR^	B^+^F^+^	W	G2	T1
P11-33	H3	SDHI^R^ AP^R^ QoI^S^ Fen^HR^ FLu^LR^	B^+^F^+^	W	G2	T1
P11-14	H4	SDHI^R^ AP^S^ QoI^R^ Fen^HR^ FLu^S^	B^+^F^−^	W	G2	T2
P18-4	H5	SDHI^R^ AP^S^ QoI^R^ Fen^HR^ FLu^S^	B^−^F^+^	W	G2	T2
P18-45	H6	SDHI^S^ AP^S^ QoI^S^ Fen^LR^ Flu^HR^	B^+^F^−^	W	G2	T1
P15-21	H7	SDHI^S^ AP^S^ QoI^S^ Fen^LR^ FLu^LR^	B^+^F^−^	W	G2	T1
P18-14	H8	SDHI^S^ AP^S^ QoI^S^ Fen^LR^ FLu^LR^	B^+^F^+^	W	G2	T1
P15-36	H9	SDHI^S^ AP^R^ QoI^S^ Fen^LR^ FLu^LR^	B^+^F^−^	W	G2	T1
P16-17	H10	SDHI^S^ AP^R^ QoI^S^ Fen^LR^ FLu^LR^	B^+^F^+^	W	G2	T1
P6-9	H11	SDHI^R^ AP^R^ QoI^S^ Fen^LR^ FLu^LR^	B^+^F^−^	W	G2	T1
P5-9	H12	SDHI^R^ AP^R^ QoI^S^ Fen^LR^ FLu^S^	B^+^F^+^	W	G2	T1
P12-24	H13	SDHI^R^ AP^S^ QoI^R^ Fen^LR^ FLu^S^	B^+^F^−^	W	G2	T2
P15-2	H14	SDHI^S^ AP^S^ QoI^S^ Fen^S^ FLu^lR^	B^+^F^−^	W	G2	T1
P10-8	H15	SDHI^S^ AP^S^ QoI^R^ Fen^S^ FLu^lR^	B^+^F^+^	W	G2	T2
P10-1	H16	SDHI^S^ AP^R^ QoI^S^ Fen^S^ FLu^lR^	B^+^F^−^	W	G2	T1
P14-2	H17	SDHI^R^ AP^S^ QoI^S^ Fen^S^ FLu^lR^	B^−^F^+^	W	G1	T1
P4-5	H18	SDH^IR^ AP^S^ QoI^R^ Fen^S^ FLu^lR^	B^+^F^−^	W	G2	T2
P14-5	H19	SDHI^R^ AP^R^ QoI^R^ Fen^S^ FLu^lR^	B^+^F^−^	W	G2	T2
P15-7	H20	SDHI^S^ AP^S^ QoI^S^ Fen^S^ FLu^S^	B^+^F^−^	W	G2	T1
P11-4	H21	SDHI^R^ AP^R^ QoI^R^ Fen^S^ FLu^lR^	B^+^F^+^	W	G2	T2
P18-44	H22	SDHI^S^ AP^S^ QoI^S^ Fen^S^ FLu^S^	B^+^F^+^	W	G2	T1
P13-8	H23	SDHI^S^ AP^S^ QoI^S^ Fen^S^ FLu^S^	B^−^F^−^	W	G2	T1
P3-1	H24	SDHI^S^ AP^S^ Qo^IR^ Fen^S^ FLu^S^	B^+^F^−^	W	G2	T2
P16-15	H25	SDHI^S^ AP^S^ Qo^IR^ Fen^S^ FLu^S^	B^+^F^+^	W	G2	T2
P11-16	H26	SDHI^S^ AP^S^ Qo^IR^ Fen^S^ FLu^S^	B^−^F^+^	W	G2	T2
P18-49	H27	SDHI^S^ AP^S^ Qo^IR^ Fen^S^ FLu^S^	B^−^F^−^	W	G2	T2
P1-2	H28	SDHI^S^ AP^R^ QoI^S^ Fen^S^ FLu^S^	B^+^F^−^	W	G2	T1
P18-22	H29	SDHI^S^ AP^R^ QoI^S^ Fen^S^ FLu^S^	B^+^F^+^	W	G2	T1
P11-10	H30	SDHI^S^ AP^R^ QoI^S^ Fen^S^ FLu^S^	B^−^F^+^	W	G2	T1
P14-22	H31	SDHI^R^ AP^S^ QoI^S^ Fen^S^ FLu^S^	B^+^F^−^	W	G2	T1
P12-14	H32	SDHI^S^ AP^R^ QoI^R^ Fen^S^ FLu^S^	B^+^F^+^	W	G2	T2
P18-36	H33	SDHI^R^ AP^R^ QoI^S^ Fen^S^ FLu^S^	B^+^F^+^	W	G2	T1
P8-3	H34	SDHI^R^ AP^R^ QoI^S^ Fen^S^ FLu^S^	B^−^F^+^	W	G2	T1
P6-11	H35	SDHI^R^ AP^S^ QoI^R^ Fen^S^ FLu^S^	B^+^F^−^	W	G2	T2
P11-8	H36	SDHI^R^ AP^S^ QoI^R^ Fen^S^ FLu^S^	B^+^F^+^	W	G2	T2
P3-8	H37	SDHI^R^ AP^S^ QoI^R^ Fen^S^ FLu^S^	B^−^F^+^	W	G2	T2
P17-2	H38	SDHI^R^ AP^R^ QoI^R^ Fen^S^ FLu^S^	B^+^F^−^	W	G2	T2
P4-4	H39	SDHI^R^ AP^R^ QoI^R^ Fen^S^ FLu^S^	B^−^F^+^	W	G2	T2
P16-31	H40	SDHI^R^ AP^R^ QoI^R^ Fen^HR^ FLu^LR^	B^+^F^+^	W	G2	T2
P13-5	H41	SDHI^R^ AP^R^ QoI^R^ Fen^HR^ FLu^LR^	B^−^F^+^	W	G2	T2
P18-9	H42	SDHI^R^ AP^R^ QoI^R^ Fen^HR^ FLu^S^	B^+^F^+^	W	G2	T2
P8-9	H43	SDHI^R^ AP^R^ QoI^R^ Fen^HR^ FLu^S^	B^−^F^+^	W	G2	T2
P17-10	H44	SDHI^R^ AP^S^ QoI^R^ Fen^HR^ FLu^LR^	B^+^F^+^	W	G2	T2
P16-26	H45	SDHI^S^ AP^R^ QoI^R^ Fen^LR^ FLu^LR^	B^+^F^+^	W	G2	T1
P8-6	H46	SDHI^R^ AP^S^ QoI^S^ Fen^HR^ FLu^S^	B^−^F^+^	W	G2	T1
P18-55	H47	SDHI^S^ AP^R^ QoI^R^ Fen^LR^ FLu^LR^	B^+^F^+^	W	G2	T2
P2-1	H48	SDHI^S^ AP^R^ QoI^R^ Fen^HR^ FLu^S^	B^+^F^+^	W	G2	T2
P15-26	H49	SDHI^S^ AP^R^ QoI^S^ Fen^LR^ FLu^S^	B^+^F^+^	W	G2	T1
P16-19	H50	SDHI^S^ AP^S^ QoI^R^ Fen^LR^ FLu^LR^	B^+^F^−^	W	G2	T2
P13-15	H51	SDHI^S^ AP^S^ QoI^R^ Fen^LR^ FLu^LR^	B^−^F^+^	W	G2	T2
P12-3	H52	SDHI^S^ AP^S^ QoI^R^ Fen^LR^ FLu^LR^	B^−^F^−^	W	G2	T2
P14-27	H53	SDHI^S^ AP^S^ QoI^R^ Fen^LR^ FLu^S^	B^+^F^+^	W	G2	T2
P18-33	H54	SDHI^S^ AP^S^ QoI^R^ Fen^LR^ FLu^S^	B^−^F^+^	W	G2	T2
P11-17	H55	SDHI^S^ AP^S^ QoI^R^ Fen^LR^ FLu^S^	B^+^F^+^	W	G2	T1
P2-2	H56	SDHI^S^ AP^S^ QoI^S^ Fen^HR^ Flu^HR^	B^−^F^+^	W	G2	T1
P18-11	H57	SDHI^S^ AP^S^ QoI^S^ Fen^LR^ FLu^S^	B^+^F^−^	W	G2	T1
P15-16	H58	SDHI^S^ AP^S^ QoI^S^ Fen^LR^ FLu^S^	B^−^F^+^	W	G2	T1
P12-28	H59	SDHI^S^ AP^S^ QoI^S^ Fen^S^ FLu^S^	B^+^F^+^	S	G2	T1
P16-24	H60	SDHI^S^ AP^S^ QoI^S^ Fen^HR^ FLu^S^	B^−^F^+^	S	G2	T1

^a^ SDHI, succinate dehydrogenase inhibitor; AP, anilinopyrimidine; QoI, quinone outside inhibitor; Fen, sterol biosynthesis inhibitors (SBIs)—class III; Flu, phenylpyrroles; S, sensitivity; R, resistance; LR, low resistance; HR, high resistance. ^b^ B, *Boty*; F, *Flipper*; +, presence; −, absence. ^c^ W, wild-type sequence of *mrr1* gene; S, *mrr1* gene sequence associated with *Botrytis cinerea* group S. ^d^ G1, *Botrytis* group I; G2, *Botrytis* group II. ^e^ T1, intron in the *cytB* gene; T2, intronless *cytB* gene.

**Table 4:** Accession numbers of gene sequences of *Botrytis* strains obtained in the present study.

Isolate	Species	Origin		Accession Number			
		Host	Province	*rpb2*	*g3pdh*	*hsp60*	*nep2*
P14-2	*Botrytis sinoviticola*	Strawberry	Mazandaran	OR962124	OR962092	OR962108	na
P18-33	*Botrytis cinerea*	Strawberry	Kurdistan	OR962134	OR962102	OR962118	OR962149
P18-13	*Botrytis cinerea*	Strawberry	Kurdistan	OR962130	OR962098	OR962114	OR962145
P17-2	*Botrytis cinerea*	Roses	Khuzestan	OR962138	OR962106	OR962122	OR962152
P14-27	*Botrytis cinerea*	Strawberry	Mazandaran	OR962135	OR962103	OR962119	OR962150
P16-15	*Botrytis cinerea*	Strawberry	Isfahan	OR962131	OR962099	OR962115	OR962146
P15-7	*Botrytis cinerea*	Strawberry	Kerman	OR962128	OR962096	OR962112	OR962143
P8-3	*Botrytis cinerea*	Roses	Gilan	OR962126	OR962094	OR962110	OR962141
P6-11	*Botrytis cinerea*	Roses	Hamedan	OR962137	OR962105	OR962121	OR962151
P10-8	*Botrytis cinerea*	Roses	Fars	OR962123	OR962091	OR962107	OR962139
P15-16	*Botrytis cinerea*	Strawberry	Kerman	OR962129	OR962097	OR962113	OR962144
P18-55	*Botrytis prunorum*	Strawberry	Kurdistan	OR962127	OR962095	OR962111	OR962142
P18-22	*Botrytis prunorum*	Strawberry	Kurdistan	OR962125	OR962093	OR962109	OR962140
P18-45	*Botrytis prunorum*	Strawberry	Kurdistan	OR962133	OR962101	OR962117	OR962148
P16-19	*Botrytis prunorum*	Strawberry	Isfahan	OR962132	OR962100	OR962116	OR962147
P8-9	*Botrytis prunorum*	Roses	Gilan	OR962136	OR962104	OR962120	na

## Data Availability

All the sequence data generated in this study are publicly available from the NCBI/GenBank database (http://www.ncbi.nlm.nih.gov (accessed on 1 October 2024)) with the accession numbers listed in Table 4.

## Supplementary Materials

The following supporting information can be downloaded at: https://www.mdpi.com/article/10.3390/jof10110764/s1, Table S1: Codes of sequences of *rpb2*, *hsp60*, *g3pdh*, and *nep2* genes from *Botrytis* species used in phylogenetic tree preparation; Table S2: Mean disease severity ± standard error on strawberry fruits and cucumber cotyledons at different days after inoculation (DAI) with *Botrytis sinoviticola* strain P14-2, three strains of *Botrytis prunorum* (P18-45, P16-19, and P8-9), and three strains of *Botrytis cinerea* (P18-13, P15-7, and P6-11); Table S3: Genetic and phenotypic data for 345 *Botrytis isolates* in this study; Figure S1: Single-gene *rpb2* (A) and *nep2* (B) phylogenetic trees prepared by using Mega7 software [46,47,48,49,50,51,52,53,54,55,56,57].

## References

[1] FAOSTAT. Food and Agriculture Organization (2019). The Database of Annual Production. FAOSTAT. Statistical Database. https://www.fao.org/faostat/en/#data/QC.

[2] Nabizadeh H., Ahmadpour A., Gosta Y. (2022). Study on the diversity of *Botrytis* spp. from different plants in West Azarbaijan province and Sanadaj city (Kurdistan province). Iran. J. Plant Pathol..

[3] Kulkarni S.J., Arafat M.Y., Saleem I., Ali J., Khan A., Balhareth H.H. (2022). An insight into research and investigations of gray mold focused on *Botrytis cinerea*. Driving Factors for Venture Creation and Success in Agricultural Entrepreneurship.

[4] Gül E., Karakaya A., Ergül A. (2023). Determination of the frequency and virulence of some *Botrytis cinerea* isolates and a new *Botrytis prunorum* cryptic species causing grey mould disease on greenhouse tomatoes. Plant Pathol..

[5] Maia J.N., Beger G., Pereira W.V., De Mio L.L.M., Duarte H.D.S.S. (2021). Gray mold in strawberries in the Paraná state of Brazil is caused by *Botrytis cinerea* and its isolates exhibit multiple-fungicide resistance. Crop Prot..

[6] Fekrikohan S., Atashi Khalilabad A., Fotouhifar K., Sharifnabi B. (2022). First report of *Botrytis cinerea* and *Alternaria alternata* on *Pelargonium grandiflorum* in Iran. Mycol. Iran..

[7] Naeimi S., Zare R. (2013). Evaluation of indigenous *Trichoderma* spp. isolates in biological control of *Botrytis cinerea*, the causal agent of strawberry gray mold disease. Biocontrol Plant Prot..

[8] Garfinkel A.R., Coats K.P., Sherry D.L., Chastagner G.A. (2019). Genetic analysis reveals unprecedented diversity of a globally-important plant pathogenic genus. Sci. Rep..

[9] Garfinkel A.R. (2021). The history of *Botrytis* taxonomy, the rise of phylogenetics, and implications for species recognition. Phytopathology.

[10] Ziedan E.H., Attallah A.G., Abd-El-Aal S.K., Sahab A.F. (2018). Molecular identification and pathogenic potential of *Botrytis cinerea* isolates causing fruit blight of cucumber under protective greenhouse in Egypt. Plant Arch..

[11] Dean R., Van Kan J.A., Pretorius Z.A., Hammond-Kosack K.E., Di Pietro A., Spanu P.D., Rudd J.J., Dickman M., Khahmann R., Ellis J. (2012). The Top 10 fungal pathogens in molecular plant pathology. Mol. Plant Pathol..

[12] Giraud T., Fortini D., Levis C., Lamarque C., Leroux P., Lobuglio K., Brygoo Y. (1999). Two sibling species of the *Botrytis cinerea* complex, *transposa* and *vacuma*, are found in sympatry on numerous host plants. Phytopathology.

[13] Giraud T., Fortini D., Levis C., Leroux P., Brygoo Y. (1997). RFLP markers show genetic recombination in *Botryotinia fuckeliana* (*Botrytis cinerea*) and transposable elements reveal two sympatric species. Mol. Biol. Evol..

[14] Wessels B., Linde C., Fourie P., Mostert L. (2016). Genetic population structure and fungicide resistance of *Botrytis cinerea* in pear orchards in the Western Cape of South Africa. Plant Pathol..

[15] De Miccolis Angelini R.M., Rotolo C., Masiello M., Pollastro S., Ishii H., Faretra F. (2012). Genetic analysis and molecular characterisation of laboratory and field mutants of *Botryotinia fuckeliana* (*Botrytis cinerea*) resistant to QoI fungicides. Pest Manag. Sci..

[16] Staats M., van Baarlen P., Van Kan J.A. (2005). Molecular phylogeny of the plant pathogenic genus *Botrytis* and the evolution of host specificity. Mol. Biol. Evol..

[17] Staats M., Van Baarlen P., Schouten A., Van Kan J.A. (2007). Functional analysis of NLP genes from *Botrytis elliptica*. Mol. Plant Pathol..

[18] Zhang J., Zhang L., Li G.Q., Yang L., Jiang D.H., Zhuang W.Y., Huang H.C. (2010). *Botrytis sinoallii*: A new species of the grey mould pathogen on Allium crops in China. Mycoscience.

[19] Zhou Y.J., Zhang J., Wang X.D., Yng L., Jiang D.H., Li G.Q., Hsiang T., Zhuang W.Y. (2014). Morphological and phylogenetic identification of *Botrytis sinoviticola*, a novel cryptic species causing gray mold disease of table grapes (*Vitis vinifera*) in China. Mycologia.

[20] Liu Q., Li G., Li J., Chen S. (2016). *Botrytis eucalypti*, a novel species isolated from diseased Eucalyptus seedlings in South China. Mycol. Prog..

[21] Leroch M., Plesken C., Weber R.W., Kauff F., Scalliet G., Hahn M. (2013). Gray mold populations in German strawberry fields are resistant to multiple fungicides and dominated by a novel clade closely related to *Botrytis cinerea*. Appl. Environ. Microbiol..

[22] De Miccolis Angelini R.M., Habib W., Rotolo C., Pollastro S., Faretra F. (2010). Selection, characterization and genetic analysis of laboratory mutants of *Botryotinia fuckeliana* (*Botrytis cinerea*) resistant to the fungicide boscalid. Eur. J. Plant Pathol..

[23] Ma Z., Michailides T.J. (2005). Genetic structure of *Botrytis cinerea* populations from different host plants in California. Plant Dis..

[24] Habib W., De Miccolis Angelini R.M., Rotolo C., Pollastro S., Faretra F. Genetic variation in *Botryotinia fuckeliana* (*Botrytis cinerea*) populations on greenhouse vegetable crops in Lebanon. Proceedings of the Abstract Book of XVIth International Botrytis Symposium.

[25] Fournier E., Levis C., Fortini D., Leroux P., Giraud T., Brygoo Y. (2003). Characterization of *Bc-hch*, the *Botrytis cinerea* homolog of the *Neurospora crassa* het-c vegetative incompatibility locus, and its use as a population marker. Mycologia.

[26] De Miccolis Angelini R.M., Rotolo C., Masiello M., Gerin D., Pollastro S., Faretra F. (2014). Occurrence of fungicide resistance in populations of *Botryotinia fuckeliana* (*Botrytis cinerea*) on table grape and strawberry in southern Italy. Pest Manag. Sci..

[27] Eyvazi A., Massah A., Soorni A., Babaie G. (2021). Molecular phylogenetic analysis shows that causal agent of maize rough dwarf disease in Iran is closer to rice black-streaked dwarf virus. Eur. J. Plant Pathol..

[28] Kumar S., Stecher G., Tamura K. (2016). MEGA7: Molecular Evolutionary Genetics Analysis Version 7.0 for bigger datasets. Mol. Biol Evol..

[29] Rasiukevičiūtė N., Rugienius R., Šikšnianienė J.B. (2018). Genetic diversity of *Botrytis cinerea* from strawberry in Lithuania. Zemdirbyste.

[30] Johnston P.R., Hoksbergen K., Park D., Beever R.E. (2014). Genetic diversity of *Botrytis* in New Zealand vineyards and the signi–ficance of its seasonal and regional variation. Plant Pathol..

[31] Mirzaei S., Goltapeh E.M., Shams-bakhsh M. (2007). Taxonomical studies on the genus *Botrytis* in Iran. J. Agric. Technol..

[32] Ferrada E.E., Latorre B.A., Zoffoli J.P., Castillo A. (2016). Identification and characterization of *Botrytis* blossom blight of Japanese plums caused by *Botrytis cinerea* and *B. prunorum* sp. nov. in Chile. Phytopathology.

[33] Schumacher J. (2017). How light affects the life of *Botrytis*. Fungal Genet. Biol..

[34] Amiri A., Zuniga A.I., Peres N.A. (2018). Prevalence of *Botrytis* cryptic species in strawberry nursery transplants and strawberry and blueberry commercial fields in the eastern United States. Plant Dis..

[35] Isaza L., Zuluaga Y.P., Marulanda M.L. (2019). Morphological, pathogenic and genetic diversity of *Botrytis cinerea* Pers. in blackberry cultivations in Colombia. Rev. Bras. Frutic..

[36] Nielsen K.A., Skårn M.N., Strømeng G.M., Brurberg M.B., Stensvand A. (2022). Pervasive fungicide resistance in *Botrytis* from strawberry in Norway: Identification of the grey mould pathogen and mutations. Plant Pathol..

[37] Testempasis S., Puckett R.D., Michailides T.J., Karaoglanidis G.S. (2020). Genetic structure and fungicide resistance profile of *Botrytis* spp. populations causing postharvest gray mold of pomegranate fruit in Greece and California. Postharvest Biol. Technol..

[38] Saito S., Margosan D., Michailides T.J., Xiao C.L. (2016). *Botrytis californica*, a new cryptic species in the *B. cinerea* species complex causing gray mold in blueberries and table grapes. Mycologia.

[39] Esterio M., Osorio-Navarro C., Carreras C., Azócar M., Copier C., Estrada V., Auger J. (2020). *Botrytis prunorum* associated to *Vitis vinifera* blossom blight in Chile. Plant Dis..

[40] Riquelme D., Aravena Z., Valdés-Gómez H., Latorre B.A., Díaz G.A., Zoffoli J.P. (2021). Characterization of *Botrytis cinerea* and *B. prunorum* from healthy floral structures and decayed ‘Hayward’ kiwifruit during post-harvest storage. Plant Dis..

[41] Acosta Morel W., Marques-Costa T.M., Santander-Gordón D., Anta Fernández F., Zabalgogeazcoa I., Vázquez de Aldana B.R., Sukno S.A., Díaz-Mínguez J.M., Benito E.P. (2019). Physiological and population genetic analysis of *Botrytis* field isolates from vineyards in Castilla y León, Spain. Plant Pathol..

[42] Moparthi S., Parikh L.P., Gunnink Troth E.E., Burrows M.E. (2023). Identification and prevalence of seedborne *Botrytis* spp. in dry pea, lentil, and chickpea in Montana. Plant Dis..

[43] Walker A.S., Gautier A., Confais J., Martinho D., Viaud M., Le P Cheur P., Dupont J., Fournier E. (2011). *Botrytis pseudocinerea*, a new cryptic species causing gray mold in French vineyards in sympatry with *Botrytis cinerea*. Phytopathology.

[44] Plesken C., Westrich L.D., Hahn M. (2015). Genetic and phenotypic characterization of *Botrytis calthae*. Plant Pathol..

[45] Sadeghi A., Atghia O., Javan-Nikkhah M. Occurrence of *Botrytis sinoviticola* on pomegranate fruit. Proceedings of the 23rd Iranian Plant Protection Congress, Gorgan University of Agricultural Sciences and Natural Resources.

[46] Garfinkel A.R., Lorenzini M., Zapparoli G., Chastagner G.A. (2017). *Botrytis euroamericana*, a new species from peony and grape in North America and Europe. Mycologia.

[47] Zhang M., Wu H.Y., Wang X.J., Sun B. (2014). First report of *Botrytis cinerea* causing fruit rot of *Pyrus sinkiangensis* in China. Plant Dis..

[48] Garfinkel A.R., Coats K.P., Chastagner G.A. Identification of *Botrytis paeoniae* microsatellites using Ion Proton technology. Proceedings of the XII International Symposium on Flower Bulbs and Herbaceous Perennials.

[49] Chen X.R., Huang S.X., Wang H., Zhang Y., Ji Z.L. (2019). First report of *Botrytis cinerea* causing leaf spot of Chinese quince in China. Plant Dis..

[50] Harper L.A., Derbyshire M.C., Lopez-Ruiz F.J. (2019). Identification and characterization of *Botrytis medusae*, a novel cryptic species causing grey mould on wine grapes in Australia. Plant Pathol..

[51] Brauna-Morževska E., Stoddard F.L., Bankina B., Kaņeps J., Bimšteine G., Petrova I., Fridmanis D. (2023). Evaluation of pathogenicity of *Botrytis* species isolated from different legumes. Front. Plant Sci..

[52] Rupp S., Weber R.W., Rieger D., Detzel P., Hahn M. (2017). Spread of *Botrytis cinerea* strains with multiple fungicide resistance in German horticulture. Front. Microbiol..

[53] Lorenzini M., Zapparoli G. (2014). An isolate morphologically and phylogenetically distinct from *Botrytis cinerea* obtained from withered grapes possibly represents a new species of *Botrytis*. Plant pathol..

[54] Van Kan J.A., Stassen J.H., Mosbach A., Van Der Lee T.A., Faino L., Farmer A.D., Papasotiriou D.G., Zhou S., Seidl M.F., Cottam E. (2017). A gapless genome sequence of the fungus *Botrytis cinerea*. Mol. Plant Pathol..

[55] Prasannath K., Galea V.J., Akinsanmi O.A. (2023). Diversity and pathogenicity of species of *Botrytis*, *Cladosporium*, *Neopestalotiopsis* and *Pestalotiopsis* causing flower diseases of macadamia in Australia. Plant Pathol..

[56] Amselem J., Cuomo C.A., van Kan J.A., Viaud M., Benito E.P., Couloux A., Dickman M. (2011). Genomic analysis of the necrotrophic fungal pathogens *Sclerotinia sclerotiorum* and *Botrytis cinerea*. PLoS Genet..

[57] Marin M.V., Peres N.A. (2022). First Report of *Botrytis cinerea* causing leaf spot on strawberry in Florida. Plant Dis..

