# Supplementary material for: Genetic and Phenotypic Characterization of Botrytis Populations from Economic and Wild Host Plants in Iran
Source: J Fungi (Basel). 2024 Nov 2;10(11):764. doi: 10.3390/jof10110764 (PMC11595591; doi:10.3390/jof10110764)
Supplement: Supplementary file 1 [file jof-10-00764-s001.zip › Supplementary files - Table S1 and S2 Figure S1.pdf]

Supplementary Table S1. Codes of sequences of *rpb2*, *hsp60*, *g3pdh*, and *nep2* genes from *Botrytis* species used in phylogenetic tree preparation.

Code	Species	Genbank accession number				References
		rpb2	*hsp60*	*g3pdh*	*nep2*	
**BCS1**	*Botrytis aclada*	AJ745663.1	AJ716049.1	AJ704991.1	AM087087.1	[16] [17]
**BCS2**		FJ169674.1	FJ169657.1	FJ169645.1	KX364408.1	[18] [47]
**BCS3**	*Botrytis allii*	AJ745668.1	AJ716054.1	AJ704994.1	Na¥	[16]
**BCS4**		AJ745667.1	AJ716058.1	AJ704997.1	Na	[16]
**BCS5**	*Botrytis byssoidea*	AJ745670.1	AJ716059.1	AJ704998.1	AM087079.1	[17]
**BCS6**		FJ169681.1	FJ169661.1	FJ169652.1	Na	[18]
**BCS7**	*Botrytis californica*	KJ937053.1	KJ937063.1	KJ937073.1	Na	[37]
**BCS8**		KJ937052.1	KJ937062.1	KJ937072.1	Na	[37]
**BCS9**	*Botrytis calthae*	AJ745671.1	AJ716060.1	AJ704999.1	AM087088.1	[16] [17]
**,BCS10**		AJ745673.1.	AJ716062.1	AJ705001.1	KJ668046.1	[16] [43]
**BCS11**	*Botrytis cinerea*	MH796664.1	MH796663.1	MH796662.1	XM001550999.2	[48] [53]
**BCS12**		MZ356229.1	MZ344233.1	MZ344222.1	CP009806.1	[54] [55]
**BCS13**		KY275258.1	KY275257.1	KY275256.1	Na	[20]
**BCS14**		MZ288751.1	MZ288748.1	MZ288754.1	Na	[56]
**BCS15**	*Botrytis* group S	-	MH260416.1	MH277451.1	Na	[41]
**BCS16**		-	MH260420.1	MH277452.1	Na	[41]
**BCS17**	*Botrytis convoluta*	AJ745680.1	AJ716069.1	AJ705008.1	AM087062.1	[16] [17]
**BCS18**		AJ745679.1	AJ716068.1	AJ705007.1	AM087061.1	[16] [17]
**BCS19**	*Botrytis croci*	AJ745681.1	AJ716070.1	AJ705009.1	AM087065.1	[16] [17]
**BCS20**	*Botrytis draytonii*	AJ745691.1	AJ716080.1	AJ705019.1	AM087072.1	[16] [17]
**BCS21**	*Botrytis eucalypti*	KX301029.1	KX301025.1	KX301021.1	KX301037.1	[20]

**BCS22**		KX301028.1	KX301024.1	KX301020.1	KX301036.1	[20]
**BCS23**	*Botrytis elliptica*	AJ745684.1	AJ716073.1	AJ705012.1	AM087080.1	[16]
						[17]
**BCS24**		AM231321.1	AM232667.1	AM231163.1	AM087089.1	[17]
**BCS25**	*Botrytis euroamericana*	KC191679.1	KC191678.1	KC191677.1	KC762945.1	[52]
**BCS26**		KX266740.1	KX266734.1	KX266728.1	KX266752.1	[45]
**BCS27**	*Botrytis fabae*	EU563116.1	EU563098.1	EU563110.1	DQ211831.1	[18]
						[17]
**BCS28**		OP623537.1	OP623558.1	OP623579.1	DQ211832.1	[50]
						[17]
**BCS29**	*Botrytis fabiopsis*	EU514473.1	EU514482.1	EU519211.1	Na	[18]
						[17]
**BCS30**		OP623538.1	OP623559.1	OP623580.1	Na	[50]
**BCS31**	*Botrytis ficariarum*	AJ745687.1	AJ716076.1	AJ705015.1	AM087085.1	[16]
**BCS32**		AJ745687.1	AJ716077.1	AJ705016.1	Na	[17]
**BCS33**	*Botrytis fragariae*	KX429712.1	KX429694.1	KX429705.1	KX429725.1	[51]
**BCS34**		KX429708.1	KX429695.1	KX429702.1	KX429726.1	[51]
**BCS35**	*Botrytis galanthina*	AJ745690.1	AJ716078.1	AJ705017.1	AM087067.1	[16]
						[17]
**BCS36**		AJ745689.1	AJ716079.1	AJ705018.1	Na	[16]
**BCS37**	*Botrytis gladiolorum*	AJ745692.1	AJ716081.1	AJ705020.1	Na	[16]
						[17]
**BCS38**		AJ745691.1	AJ716080.1	AJ705019.1	Na	[16]
						[17]
**BCS39**	*Botrytis globosa*	AJ745693.1	AJ716083.1	AJ705022.1	AM087070.1	[16]
						[17]
**BCS40**		AJ745694.1	AJ716082.1	AJ705021.1	AM087071.1	[16]
**BCS41**	*Botrytis hyacinthi*	AJ745695.1	AJ716084.1	AJ705023.1	AM087066.1	[16]
						[17]
**BCS42**		AJ745696.1	AJ716085.1	AJ705024.1	Na	[16]
**BCS43**	*Botrytis mali*	MT604337.1	MT604533.1	MT604433.1	Na	[40]
**BCS44**		MT604336.1	MT604532.1	MT604432.1	Na	[40]
**BCS45**	*Botrytis medusae*	MH732870.1	MH732866.1	MH732861.1	MK211255.1	[49]
						[49]
**BCS46**	*Botrytis narcissicola*	AJ745697.1	AJ716087.1	AJ705026.1	AM087078.1	[16]

						[17]
**BCS47**		AJ745703.1	AJ716092.1	AJ705031.1	Na	[16]
**BCS48**	*Botrytis paeoniae*	KX266742.1	KX266736.1	KX266730.1	KX266754.1	[45]
**BCS49**		KX266741.1	KX266735.1	KX266729.1	KX266753.1	[45]
**BCS50**	*Botrytis pelargonii*	AJ745701.1	AJ716090.1	AJ705029.1	DQ211834.1	[16]
						[17]
**BCS51**	*Botrytis porri*	AJ745704.1	AJ716093.1	AJ705032.1	AM087063.1	[16]
						[17]
**BCS52**		FJ169683.1	FJ169663.1	FJ169653.1	Na	[18]
**BCS53**	*Botrytis polyblastis*	AJ745702.1	AJ716091.1	AJ705030.1	AM087074.1	[16]
						[17]
**BCS54**		AJ745703.1	AJ716092.1	AJ705031.1	AM087073.1	[16]
						[17]
**BCS55**	*Botrytis prunorum*	KP339986.1	KP339993.1	KP339979.1	KP400595.1	[32]
**BCS56**		KP339987.1	KP339994.1	KP339980.1	KP400596.1	[32]
**BCN1**		Na	Na	Na	OL743433.1	[35]
**BCN2**		Na	Na	Na	OL743434.1	[35]
**BCN3**		Na	Na	Na	KR425425.1	[32]
**BCN4**		Na	Na	Na	KR425427.1	[32]
**BCN5**		Na	Na	Na	KR425424.1	[32]
**BCN6**		Na	Na	Na	KR425426.1	[32]
**BCN7**		Na	Na	Na	MN327630.1	[38]
**BCN8**		Na	Na	Na	MN327631.1	[38]
**BCN9**		Na	Na	Na	MN327632.1	[38]
**BCS57**	*Botrytis pseudocinerea*	OP623545.1	OP623566.1	OP623587.1	OK556314.1	[50]
						[35]
**BCS58**		MH732871.1	MH732865.1	MH732860.1	OK556316.1	[49]
						[35]
**BCS59**	*Botrytis ranunculi*	AJ745706.1	AJ716095.1	AJ705034.1	AM087086.1	[16]
						[17]
**BCS60**	*Botrytis sinoallii*	EU514479.1	EU514488.1	EU519217.1	Na	[18]
**BCS61**		FJ169679.1	FJ169660.1	FJ169651.1	Na	[18]
**BCS62**	*Botrytis sinoviticola*	JN692427.1	JN692399.1	JN692413.1	Na	[46]
**BCS63**		JN692426.1	JN692398.1	JN692412.1	Na	[46]
**BCS64**	*Botrytis sphaerosperma*	AJ745708.1	AJ716096.1	AJ705035.1	AM087068.1	[16]

						[17]
**BCS65**		AJ745709.1	AJ716097.1	AJ705036.1	AM087069.1	[16]
						[17]
**BCS66**	*Botrytis squamosa*	AJ745710.1	AJ716098.1	AJ705037.1	AM087084.1	[16]
						[17]
**BCS67**		AJ745707.1	AJ716100.1	AJ705039.1	AM087083.1	[16]
						[17]
**BCS68**	*Botrytis tulipae*	AJ745713.1	AJ716102.1	AJ705041.1	AM087077.1	[16]
						[17]
**BCS69**		AJ745714.1	AJ716103.1	AJ705042.1	Na	[16]
**SS**	*Sclerotinia sclerotiorum*	AJ745716.1	AJ716048.1	AJ705044.1	Na	[16]

¥Na: not available.

Supplementary Table S2. Mean disease severity ± standard error on strawberry fruits and cucumber cotyledons at different days after inoculation (DAI) with *Botrytis sinoviticola* strain P14-2, three strains of *Botrytis prunorum* (P18-45, P16-19, and P8-9), and three strains of *Botrytis cinerea* (P18-13, P15-7, and P6-11).

**Isolate**	**Strawberry fruits**					**Cucumber cotyledons**				
	3 DAI	4 DAI	5 DAI	6 DAI	7 DAI	3 DAI	4 DAI	5 DAI	6 DAI	7 DAI
P14-2	3.5±0.9	5.9±0.9	6.7±0.1	7.0±0.0	7.0±0.0	0.0±0.0	2.7±0.3	5.0±0.0	6.0±0.0	7.0±0.0
P18-45	3.8±0.3	4.2±0.4	5.8±0.3	6.8±0.2	7.0±0.0	1.0±0.0	2.0±0.0	3.7±0.3	4.4±0.3	5.7±0.3
P16-19	3.4±0.8	4.0±0.5	5.8±0.4	6.6±0.4	7.0±0.0	2.0±1.2	3.3±1.7	4.0±0.0	4.7±2.3	6.0±1.0
P8-9	3.6±0.8	4.8±1.0	6.2±0.7	7.0±0.0	7.0±0.0	3.7±0.3	5.7±0.3	7.0±0.0	7.0±0.0	7.0±0.0
P18-13	3.2±0.8	3.8±0.4	5.7±0.6	6.4±0.4	7.0±0.0	1.3±0.7	1.7±0.9	2.0±1.2	2.0±1.2	3.3±2.0
P15-7	3.8±0.2	4.8±0.2	5.8±0.4	7.0±0.0	7.0±0.0	0.3±0.3	0.7±0.7	1.7±1.7	2.3±2.3	2.3±2.3
P6-11	3.6±0.5	5.0±0.7	6.0±0.4	6.8±0.4	7.0±0.0	3.7±0.9	5.3±0.7	7.0±0.0	7.0±0.0	7.0±0.0

(A)

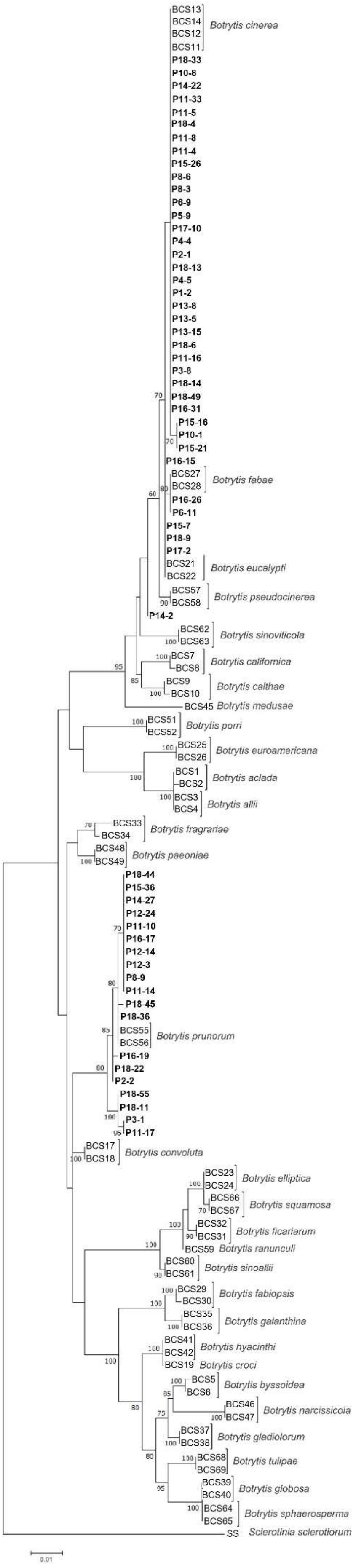
BCS13
BCS14
BCS12
BCS11
Botrytis cinerea
P18-33
P10-8
P14-22
P11-33
P11-5
P18-4
P11-8
P11-4
P15-26
P8-6
P8-3
P6-9
P5-9
P17-10
P4-4
P2-1
P18-13
P4-5
P1-2
P13-8
P13-5
P13-15
P18-6
P11-16
P3-8
P18-14
P18-49
P16-31
P15-16
P10-1
P15-21
P16-15
BCS27
BCS28
Botrytis fabae
P16-26
P6-11
P15-7
P18-9
P17-2
BCS21
BCS22
Botrytis eucalypti
BCS57
BCS58
Botrytis pseudocinerea
P14-2
BCS62
BCS63
Botrytis sinoviticola
BCS7
BCS8
Botrytis californica
BCS9
BCS10
Botrytis calthae
BCS45
Botrytis medusae
BCS51
BCS52
Botrytis porri
BCS25
BCS26
Botrytis euroamericana
BCS1
BCS2
Botrytis aclada
BCS3
BCS4
Botrytis allii
BCS33
BCS34
Botrytis fragrariae
BCS48
BCS49
Botrytis paeoniae
P18-44
P15-36
P14-27
P12-24
P11-10
P16-17
P12-14
P12-3
P8-9
P11-14
P18-45
P18-36
BCS55
BCS56
Botrytis prunorum
P16-19
P18-22
P2-2
P18-55
P18-11
P3-1
P11-17
BCS17
BCS18
Botrytis convoluta
BCS23
BCS24
Botrytis elliptica
BCS66
BCS67
Botrytis squamosa
BCS32
BCS31
Botrytis ficariarum
BCS59
Botrytis ranunculi
BCS60
BCS61
Botrytis sinoallii
BCS29
BCS30
Botrytis fabiopsis
BCS35
BCS36
Botrytis galanthina
BCS41
BCS42
Botrytis hyacinthi
BCS19
Botrytis croci
BCS5
BCS6
Botrytis byssoidea
BCS46
BCS47
Botrytis narcissicola
BCS37
BCS38
Botrytis gladiolorum
BCS68
BCS69
Botrytis tulipae
BCS39
BCS40
Botrytis globosa
BCS64
BCS65
Botrytis sphaerosperma
SS
Sclerotinia sclerotiorum
0.01

(B)

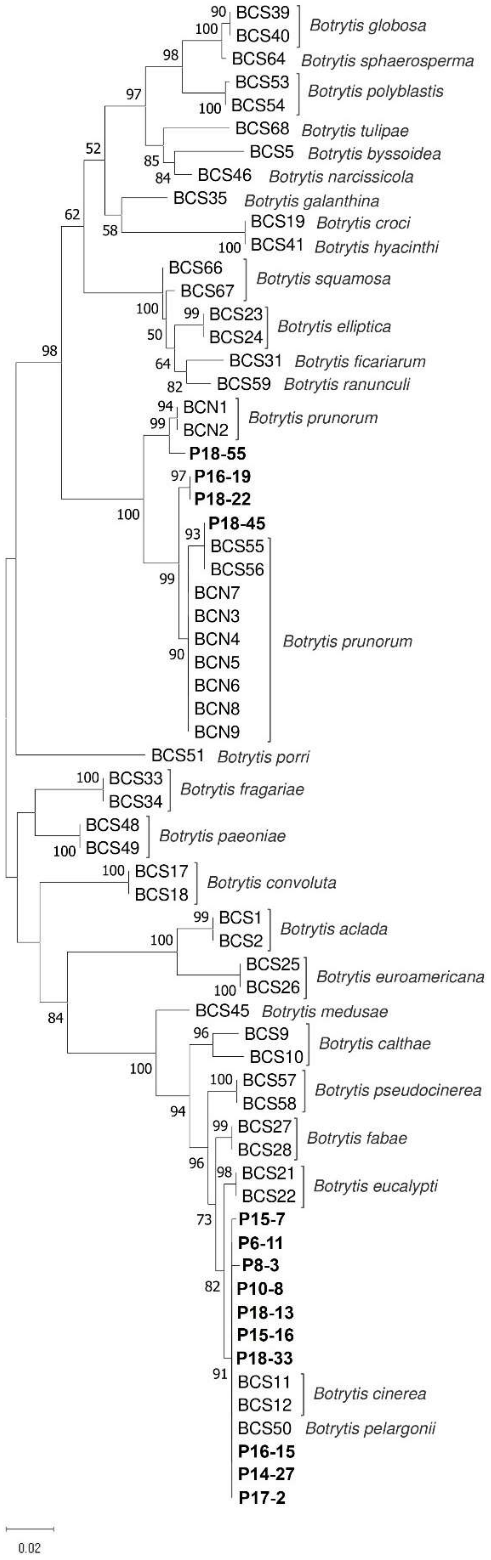

Supplementary Figure S1. Single-gene *rpb2* (A) and *nep2* (B) phylogenetic trees prepared by using Mega7 software. Iranian strains from current study are in bold. The trees are drowned to scale, with branch lengths measured in the number of substitutions per site. Bootstrap values > 60 based on 1000 replicates are shown.
